# Banxia-Houpu decoction diminishes iron toxicity damage in heart induced by chronic intermittent hypoxia

**DOI:** 10.1080/13880209.2022.2043392

**Published:** 2022-03-14

**Authors:** Ji-Xian Song, Ya-Shuo Zhao, Ya-Qin Zhen, Xin-Yue Yang, Qi Chen, Ji-Ren An, En-Sheng Ji

**Affiliations:** a Department of Physiology, Institute of Basic Medicine, Hebei University of Chinese Medicine, Shijiazhuang, PR China; b Experimental Center, Hebei University of Chinese Medicine, Shijiazhuang, PR China; c First Clinical College, Liaoning University of Traditional Chinese Medicine, Shenyang, PR China

**Keywords:** Obstructive sleep apnoea, cardiac damage, mitochondrial disfunction, rosmarinic acid

## Abstract

**Context:**

Obstructive sleep apnoea (OSA) causes chronic intermittent hypoxia (CIH), which results in mitochondrial dysfunction and generates reactive oxygen species (ROS) in the heart. Excessive free iron could accelerate oxidative damage, which may be involved in this process. Banxia-Houpu decoction (BHD) was reported to improve the apnoea hypopnoea index in OSA patients, but the specific mechanism was still unclear.

**Objective:**

To investigate whether BHD could reduce CIH-induced heart damage by regulating iron metabolism and mitochondrial function.

**Materials and methods:**

C57BL/6N mice were randomly divided into control, CIH and BHD groups. Mice were exposed to CIH (21 − 5% O_2_, 20 times/h, 8 h/d) and administered BHD (3.51, 7.01 and 14.02 g/kg, intragastrically) for 21 d. Cardiac and mitochondrial function, iron levels, apoptosis and mitophagy were determined.

**Results:**

BHD (7.01 g/kg) significantly improved cardiac dysfunction, pathological change and mitochondrial structure induced by CIH. BHD increased the Bcl-2/Bax ratio (1.4-fold) and inhibited caspase 3 cleavage in CIH mice (0.45-fold). BHD activated mitophagy by upregulating Parkin (1.94-fold) and PINK1 (1.26-fold), inhibiting the PI3K-AKT-mTOR pathway. BHD suppressed ROS generation by decreasing NOX2 (0.59-fold) and 4-HNE (0.83-fold). BHD reduced the total iron in myocardial cells (0.72-fold) and mitochondrial iron by downregulating Mfrn2 (0.81-fold) and MtFt (0.78-fold) proteins, and upregulating ABCB8 protein (1.33-fold). Rosmarinic acid, the main component of Perilla Leaf in BHD, was able to react with Fe^2+^ and Fe^3+^ *in vitro*.

**Discussion and conclusions:**

These findings encourage the use of BHD to resist cardiovascular injury and provide the theoretical basis for clinical treatment in OSA patients.

## Introduction

Obstructive sleep apnoea (OSA), which manifests with apnoeas or hypopneas, is a prevalent sleep disorder characterized by recurrent occlusion of the upper airway during sleep, leading to chronic intermittent hypoxia (CIH). A growing number of clinical studies have demonstrated that OSA was associated with an increased risk of various cardiovascular diseases, such as hypertension, coronary heart disease, myocardial hypertrophy and heart failure, thus posing a significant threat to the population (Benjafield et al. 2019; Roche et al. 2021). Animal studies have shown that CIH exposure could induce myocardial damage, ventricular systolic and diastolic dysfunction, as well as result in increased mortality of heart failure in animal models (Guan, Sun, Luo, et al. 2019; Guan, Sun, Wang, et al. 2019; Zhao et al. 2019).

Mitochondria are highly abundant in the heart and provide more than 90% of the energy supply *via* the electron transport chain (ETC) and oxidative phosphorylation (Lesnefsky et al. 2016). It is well known that iron metabolism and mitochondrial function are closely related. Serum iron enters cardiomyocytes mainly through the transferrin/transferrin receptor 1 (Tf/TfR1) system, and slightly through divalent metal transporter 1 (DMT1), calcium channels or zinc transporters (Ravingerova et al. 2020). In cardiomyocytes, most of the iron is transferred into mitochondria by mitoferrin-2 (Mfrn2) to exert an effect on the synthesis of haem or iron-sulphur clusters and ultimately participates in the ETC and oxidative phosphorylation (Paul et al. 2017; Ravingerova et al. 2020). Similar to ischaemia/reperfusion (I/R), CIH exposure may suppress the mitochondrial function and lead to the generation of reactive oxygen species (ROS), including •O_2_^−^, H_2_O_2_ and •OH^−^ (Chen and Zweier 2014; Kim et al. 2014; Zhao et al. 2019). An excess of free iron has been reported to mediate •O_2_^−^ and H_2_O_2_ to produce the most toxic •OH^−^ by the Fenton and Haber − Weiss reaction (Chang et al. 2016). Clinical data has indicated a higher level of oxidative stress markers (DeMartino et al. 2016) and a lower number of mitochondrial DNA copies (Lacedonia et al. 2015) in OSA patients proportional to the extent of the disease. Coincidentally, an animal study confirmed that I/R could increase iron levels in the mitochondria of heart tissues, whereas a specific iron chelator could relieve mitochondrial function and improve ventricular remodelling (Chang et al. 2016). Our previous studies have revealed the presence of iron deposition in the brain (An et al. 2020) and kidney after CIH exposure (Guan, Sun, Luo, et al. 2019; Guan, Sun, Wang, et al. 2019). Therefore, whether iron is linked to CIH-induced cardiac injury and the related pathological changes remains to be further explored.

Banxia-Houpu decoction (BHD) is a traditional Chinese medicine (TCM) developed by Zhang Zhongjing, and consists of Pinellia Tuber [*Pinellia ternata* (Thunb.) Breit. (Araceae)], Magnolia Cortex [*Magnolia officinalis* Rehd.et Wils. (Magnoliaceae)], Poria [*Poria cocos* (Schw.) Wolf. (Polyporaceae)], Ginger Rhizome [*Zingiber officinale* Rosc. (Zingiberaceae)] and Perilla Leaf [*Perilla frutescens* (L.) Britt. (Lamiaceae)] (Kagohashi et al. 2016). It is widely used in the treatment of functional dyspepsia (Oikawa et al. 2009), depression (Jia et al. 2017; Chen et al. 2020) and chronic obstructive pulmonary disease (Li and Bai 2019). In addition to adjusting lipid metabolism disorders and endothelial damage in OSA patients, clinical studies have also illustrated that BHD could significantly improve the severity of OSA through the ‘Phlegm-Qi’ interaction (Fang et al. 2018; Lian et al. 2020; Jiang et al. 2021). Nevertheless, it remains unclear whether BHD is involved in treating of cardiac dysfunction induced by OSA and the underlying mechanism has yet to be elucidated.

Iron is an essential trace element in vital physiological functions in humans and is found in functional forms in haemoglobin, myoglobin, cytochrome and various nonheme enzymes (Anderson and Shah 2013). The understanding of ‘Qi’ in Chinese medicine is that ‘Qi is the commander of blood’, which is typically expressed as ‘Qi can produce blood’. Therefore, there may be a relationship between iron metabolism and Chinese medicine’s ‘Qi’ theory. Moreover, iron overload may be harmful, leading to oxidative stress damage and inhibition of autophagy (Wu et al. 2016; Guan, Sun, Luo, et al. 2019; Guan, Sun, Wang, et al. 2019). For these reasons, we attempt to elucidate the role of BHD in regulating iron metabolism abnormalities in the heart induced by CIH exposure to provide new ideas for the clinical prevention and treatment of OSA.

## Materials and methods

### Preparation and determination of BHD

BHD consists of five herbal medicines, including Pinellia Tuber (12 g), Magnolia Cortex (9 g), Poria (12 g), Perilla Leaf (6 g) and Ginger Rhizome (15 g) (Table 1). Above-mentioned herbs were purchased from Shen-Wei Pharmaceutical Group Co., Ltd. The voucher specimens, identified by Professor Yu-Guang Zheng, have been deposited at Hebei University of Chinese Medicine, Shijiazhuang, China. First, these herbs were immersed in a 10 times volume of distilled water for 30 min and then decocted at boiling temperature for 40 min to get the filtrate. Then, the residues were added to an 8-fold volume of distilled water and decocted for 30 min in a simmer to get the filtrate. The filtrates of the two times were merged and centrifuged to obtain the supernatants, finally concentrated to 100 mL, which produced the BHD. This solution was stored at 4 °C.

**Table 1.** Prescription of Banxia-Houpu decoction.

Local name	Ban Xia	Hou Pu	Fu Ling	Sheng Jiang	Su Ye
Part used	Root	Bark	Sclerotium	Rhizome	Leaf
English name	Pinellia Tuber	Magnolia Cortex	Poria	Ginger Rhizome	Perilla Leaf
Latin name	*Pinellia ternata* (Thunb.) Breit., Araceae	*Magnolia officenalis* Rehd.et Wils, Magnoliaceae	*Poria cocos* (Schw.) Wolf., Polyporaceae	*Zingiber officeinale* Rosc., Zingiberaceae	*Perilla frutescens* (L.) Britt., Lamiaceae
Amount (g)	12	9	12	9	6

The qualitative and quantitative analysis of components in the aqueous extract of BHD was conducted by liquid chromatography-tandem mass spectrometry (LC-MS/MS) method. The appropriate amount of reference materials of magnolol, honokiol, succinic acid, rosmarinic acid and 6-gingerol was precisely weighed, dissolved with methanol and diluted to the corresponding reference reserve solution. Chromatographic separation was performed on a Shim-pack GIST C18 column (2.1 mm × 100 mm, 2 μm). The temperature of the column was maintained at 35 °C, the flow rate and injection volumes were 0.3 mL/min and 1 µL, respectively. The mobile phase was prepared by using acetonitrile (A) and 0.1% formic acid (B) with the gradient elution method (0–3 min 2% A, 3–6 min 2–35% A, 6–9 min 35–50% A, 9–13 min 50–75% A, 13–14.5 min 75% A and 14.5–15 min 75–2% A). The accurate mass spectrometric experiment was operated in the ESI negative mode of a Triple TOF 4600 mass spectrometer system. The following operation parameters were used: ion source gas 1 and ion source gas 2, 345 kPa; curtain gas, 241 kPa; ion spray voltage floating, 4500 V; Temperature, 550 °C; and Collision energy, 16–40 V.

### Reagents

Reagents: RIPA lysate (Servicebio, Wuhan, China; G2002) , protease inhibitor (Thermo Scientific, Waltham, MA; A32955), Tween-20 (Solarbio, Beijing, China; 9005-64-5), BCA kit (CoWin Biosciences, Cambridge, MA; CW0014), potassium ferrocyanide (Sigma-Aldrich, St. Louis, MO; 13425), ammonium ferrous sulphate (Sigma-Aldrich, St. Louis, MO; 203505), ferric trichloride (Sigma-Aldrich, St. Louis, MO; 451649), TUNEL kit (Vazyme Biotech, Nanjing, China; A112) and dihydroethidium (DHE, Cayman Chemical, Ann Arbor, MI; 19709). Antibodies: Bcl-2 (Immunoway, Plano, TX; YT0470), Bax (Servicebio, Wuhan, China; GB11007), caspase 3 (Cell Signalling Technology, Danvers, MA; #14220), Dynamin-related protein 1 (Drp1, Zen-bioscience, Durham, NC; 382977), Optic atrophy 1 (Opa1, Zen-bioscience, Durham, NC; 382025), Parkin (Boster, Pleasanton, CA; BM4909), PINK1 (Affinity, West Bridgford, UK; DF7742), LC3B (BD Bioscience, Franklin Lakes, NJ; L7543), Beclin1 (Servicebio, Wuhan, China; GB13228-1), p62 (Boster, Pleasanton, CA; PB0458), PI3K (Proteintech, Rosemont, IL; 20584-1-AP), AKT (Proteintech, Rosemont, IL; 10176-2-AP), p-AKT (Proteintech, Rosemont, IL; 66444-1-Ig), p-mTOR (Cell Signalling Technology, Danvers, MA; #9198), mTOR (Cell Signalling Technology, Danvers, MA; #9197), Nicotinamide adenine dinucleotide phosphate oxidase 2 (NOX2, GeneTex, Irvine, CA; GTX56278), 4-hydroxynonenal (4-HNE, Arigo Biolaboratories, Hsinchu, Taiwan; ARG70025), Transferrin receptor 1 (TfR1, Invitrogen, Carlsbad, CA; 13-6800), DMT1(−ire 1), Alpha Diagnostic International, NRAMP23-A, DMT1(+ire), Alpha Diagnostic International, NRAMP21-S), Ferroportin 1 (FPN1, Alpha Diagnostic International, San Antonio, TX; MTP11-A), Mfrn2 (Novus, St. Louis, MO; NBP1-59562), Mitochondrial ferritin (MtFt, Abcam, Cambridge, UK; ab66111) and ABCB8 (Abclonal, Woburn, MA; A2653), GAPDH (Servicebio, Wuhan, China; GB15002) and α-Tubulin (GeneTex, Irvine, CA; GTX628802).

### Animals

C57BL/6N mice (SPF grade, male, 20–22 g) were purchased from Beijing Vital River Laboratory Animal Technology Co., Ltd, Beijing, China. All the mice were transferred to the Experimental Animal Centre under SPF conditions and acclimated for a minimum of 1 week before experimentation. The experimental animal procedures were conducted following the National Institutes of Health Guide for the Care and Use of Laboratory Animals and approved by the Animal Care and Use Committee of Medical Ethics of Hebei University of Chinese Medicine (Animal Ethics Number, DWLL2018008).

C57BL/6N mice (*n* = 45) were randomly divided into three groups: the Normoxia group (Con), the CIH group (CIH) and the BHD group. The CIH rodent model was established to simulate OSA. The mice were placed in a controlled hypoxic chamber (OxyCycler, BioSpherix Ltd., Parish, NY) in which the O_2_ concentration was reduced from 21 to 5% over an initial period of 1.5 min and then returned to 21% over another period of 1.5 min. The cycle was repeated every 3 min for 8 h each day (9:00–17:00). The mice in the Con group were placed in the same chamber with normal air. The CIH and BHD groups mice were exposed to CIH for 21 d. In addition, before exposure each day, the mice in BHD group were administered with different measurements of BHD (3.51, 7.01 and 14.02 g/kg) by intragastric administration. Meanwhile, the mice in the Con and CIH groups were given the same volume of water.

### Echocardiography

Echocardiographic analysis was performed to evaluate cardiac function using a high-resolution ultrasound imaging system with MS-250 probe (Vevo 2100, Visualsonics Inc., Toronto, Canada). First, the mice were anaesthetized with 2.5% isoflurane in a gas mixture of 5% CO_2_ and 95% O_2_. Then, the chest hair was removed with a depilatory cream. All of these measurements were performed in a blinded manner, and the methods here reference our previous study (Zhao et al. 2019). The ejection fraction (EF), fractional shortening (FS), left ventricular end-systolic volume (LVESV) and left ventricular end-diastolic volume (LVEDV) were measured using M-mode recording of the short-axis view. The maximum velocity of the mitral valve (MV) in early diastole and systole was evaluated using four-chamber echocardiography. The ratio of MV E/A was applied to reflect the changes in cardiac function.

### Pathological staining

Haematoxylin-eosin staining was used to observe the basic structure of the heart tissue. The cardiac paraffin sections were dewaxed and rehydrated in an alcohol gradient. The sections were stained with haematoxylin, differentiated with hydrochloric acid ethanol, redyed with eosin, dehydrated with an alcohol gradient and vitrified with dimethylbenzene. Masson staining was performed to detect fibrosis in the cardiac tissue. After dewaxing and rehydration, the sections were successively stained with haematoxylin, hydrochloric acid ethanol, Masson blue liquid, distilled water, ponceau, phosphomolybdic acid and aniline blue. After the above steps were completed, the sections were dehydrated with 95% alcohol and anhydrous ethanol, cleared with xylene and sealed with neutral gum.

### TUNEL staining

TUNEL staining was applied to evaluate apoptosis in heart tissue. After dewaxing and rehydration, the sections were rinsed with 0.01 M phosphate buffer solution (PBS, pH = 7.4). Protease K solution (20 μg/mL) was dripped onto the slice and incubated for 20 min at room temperature. The sections were washed with PBS, and then the balanced solution was dripped onto sections and incubated for 30 min at room temperature. Afterward, the balanced solution was suctioned off without washing. The sections were then incubated with a reaction mixture of Bright Green Labelling Mix and Recombinant TdT Enzyme for 60 min at 37 °C. After washing, the sections were stained with DAPI for 5 min at room temperature and then sealed with an anti-fluorescence quencher. The fluorescences of FITC and DAPI were detected at 488 and 460 nm, respectively. The total number of apoptotic cells was counted using Photoshop CS Version 5.0 Software (AdobeSystems Inc., California, US).

### Transmission electron microscope

The transmission electron microscope (TEM) was performed to observe mitochondrial ultrastructure. After anaesthesia, the left ventricle tissue (1 mm^3^) was quickly taken and immersed in osmium tetroxide. The samples were dehydrated through ethanol and xylene gradients, then infiltrated, embedded and polymerized into the resin. The resin was sliced into ultrathin sections and stained with uranyl acetate and lead nitrate. The images were observed under a HITACHI HT7800 electron microscope (Hitachi High-Technologies Corp., Tokyo, Japan).

### DHE staining

The cryosections of cardiac tissue were brought to room temperature and then washed with PBS. The sections were incubated with a fluorescence quenching agent to remove the background fluorescence. Then, DHE (5 μM) was added to the slices and incubated at 37 °C for 30 min. After washing, the sections were stained with DAPI for 5 min at room temperature. Finally, the sections were sealed with an anti-fluorescence quencher.

### Western blot

First, precooled PIRA lysate was added to the myocardial tissue to prepare a homogenate (10% g/v). After centrifugation, the supernatant was collected and the protein concentration was measured using the BCA method. Total proteins were separated utilizing SDS-PAGE electrophoresis and then transferred to polyvinylidene fluoride (PVDF) membranes. The blots were sealed with 5% skimmed milk powder. The blots were then incubated with primary antibodies against Opa1, Drp1, Bcl-2, Bax, caspase 3, Parkin, PINK1, LC-3B, Beclin1, p62, PI3K, p-AKT, AKT, p-mTOR, mTOR, NOX2, 4-HNE, TfR1, DMT1(-ire), DMT1(+ire), FPN1, Mfrn2, MtFt, ABCB8, GAPDH or α-Tubulin at 4 °C overnight. The following day, the blots were incubated with HRP-conjugated secondary antibodies for 2 h at room temperature. Finally, the immunoreactive proteins were imaged by the enhanced chemiluminescence (ECL) method. ImageJ software (Rawak Software Inc., Stuttgart, Germany) was used to analyse the mean grey value of the target bands.

### Analysis of interaction with iron ions

First, the magnolol, honokiol, succinic acid, rosmarinic acid, and 6-gingerol were dissolved in 2.0 mmol/mL methanol, respectively, and then diluted with purified water to a concentration of 1.0 mmol/mL. Ammonium ferrous sulphate and ferric trichloride were dissolved individually in purified water at a 1.0 mmol/mL concentration. The BHD components solution (0.5 mL) were removed, placed into a volumetric flask (10 mL), and then diluted to 2.0 mL with purified water, respectively. Next, 2.0 mL of HAc-NAc buffer solutions (pH = 6.0) and 1.0 mL of Fe^2+^ or Fe^3+^ solutions were added and diluted to a final volume of 10 mL with pure water. After quiescence in the dark for 10 min, the mixture and single component were detected separately using a full wavelength scanning. The *λ* absorption spectra before and after reaction with Fe^2+^ and Fe^3+^ were recorded at 200–900 nm.

### Statistical analysis

All experimental data were analysed using SPSS version 23.0 statistical software (SPSS Inc., Chicago, IL), and the data are expressed as the mean ± SEM. A one-way ANOVA was performed, followed by an LSD *post hoc* test. A *p* < 0.05 was considered significant. The statistical charts were made using GraphPad Prism version 8.0 software (GraphPad Software, Inc., La Jolla, CA).

## Results

### Identification and quantitative analysis of components in BHD

**Figure 1.**
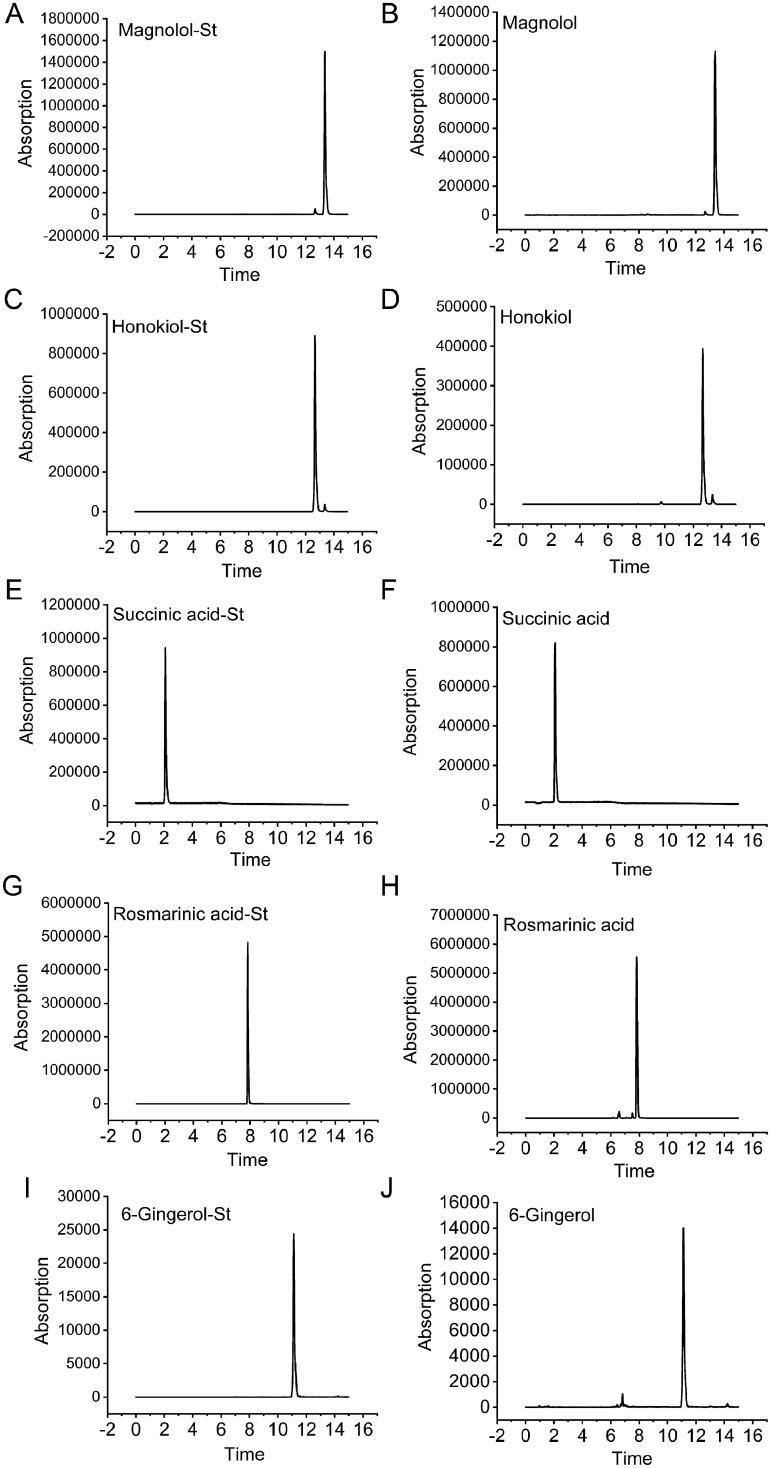
Quantitative analysis of components in the aqueous of Banxia Houpu decoction by LC-MS/MS. (A,B) The magnolol of standards (St) and BHD. (C,D) The honokiol of standards (St) and BHD. (E,F) The succinic acid of standards (St) and BHD. (G,H) The rosmarinic acid of standards (St) and BHD. (I,J) The 6-gingerol of standards (St) and BHD.

Figure 1 shows the qualitative analysis of components in the aqueous extract of BHD as analysed by LC-MS/MS. The aqueous extract contained 1.97 μg/mL magnolol, 1.32 μg/mL honokiol, 5.72 μg/mL succinic acid, 18.83 μg/mL rosmarinic acid and 4.49 μg/mL 6-gingerol.

### BHD improved cardiac dysfunction and histological changes induced by CIH

**Figure 2.**
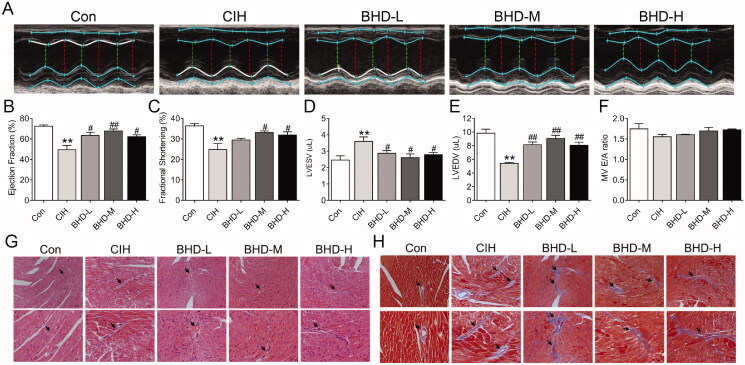
Cardiac dysfunction induced by CIH exposure in mice. (A) M-model echocardiography in mice (*n* = 6). (B) The ejection fraction (EF) of the left ventricle (*n* = 6). (C) The fractional shortening (FS) (*n* = 6). (D) The left ventricular end-systolic volume (LVESV, *n* = 6). (E) The left ventricular end-diastolic volume (LVEDV, *n* = 6). (F) The velocity ratio of the E peak to the A peak in the cardiac mitral valve (MV E/A) (*n* = 6). (G,H) The H&E staining and Masson’s trichrome staining of the Con, CIH, BHD-L, BHD-M and BHD-H groups (scale bar = 20 μm, *n* = 3). The data are presented as the mean ± SEM. **p < 0.01 *vs.* Con group. *^#^p* < 0.05, *^##^p* < 0.01 *vs.* CIH group.

We first established a CIH mice model and evaluated systolic and diastolic function using M-model echocardiography (Figure 2(A)). Compared with normal mice, the left ventricular EF and short-axis FS of CIH mice were both decreased (Figure 2(B,C)). The mean value of LVESV was elevated compared with that of the Con group (Figure 2(D)). These results indicated that CIH induced systolic dysfunction, and in contrast, BHD treatment improved this dysfunction (Figure 2(B–D)) especially, intermediate doses of BHD showed the best therapeutic effect. In parallel, we evaluated the diastolic function of mice. The value of LVEDV decreased in CIH mice (Figure 2(E)); however, the ratio of E/A did not alter between CIH mice and Con mice (Figure 2(F)). The decreased LVEDV in CIH mice significantly improved after BHD treatment at each dose (Figure 2(E)).

The images from H&E staining showed that the cardiomyocytes of CIH mice were swollen and showed a disordered arrangement accompanied by inflammatory infiltration compared with those of normal mice (Figure 2(G)). The BHD treatment improved the changes in the myocardial structure induced by CIH exposure. Masson staining was applied to assess the myocardial fibrosis of mice, which indicated extra fibrosis in the hearts of CIH mice (Figure 2(H)). However, fibrillation improved in CIH mice treated with BHD. These images suggested that CIH exposure resulted in relatively substantial damage to cardiomyocytes; conversely, BHD significantly mitigated pathological damage. The intermediated doses of BHD showed a better therapeutic effect.

### BHD attenuated CIH-induced mitochondrial damage and apoptosis

**Figure 3.**
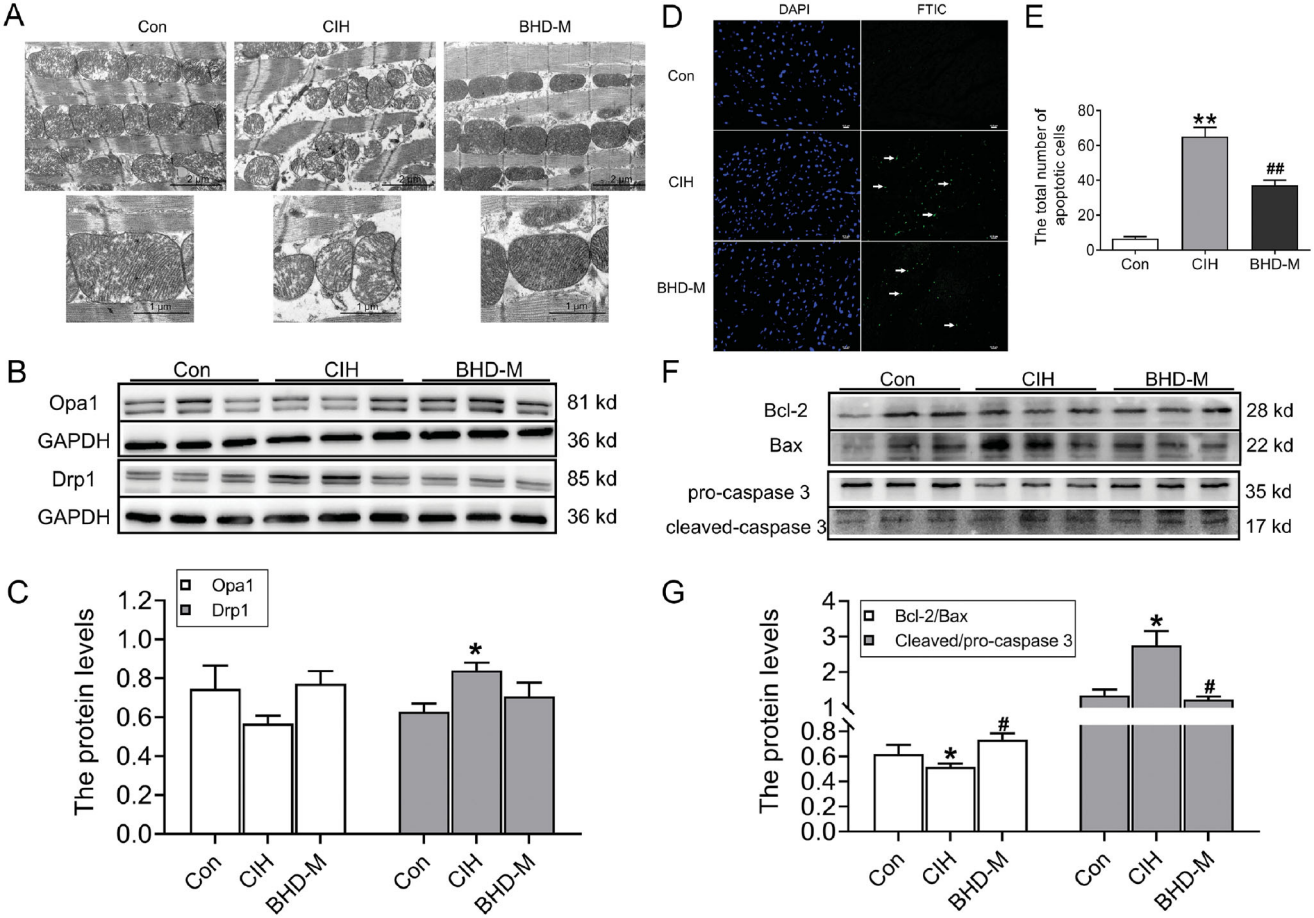
Mitochondrial damage and mitochondrial pathway-dependent apoptosis in the hearts of CIH mice. (A) The TEM images of mitochondria in the heart. (B,C) The expression of Opa1 and Drp1 proteins in heart tissue by western blot. (D) The TUNEL staining of heart tissue (scale bar = 12.5 μm, *n* = 3). (E) The total number of apoptotic cells as shown in panel D. (F,G) The expression of Bcl-2/Bax and cleaved-caspase 3/pro-caspase 3 proteins in heart tissue by western blot. The data are presented as the mean ± SEM, *n* = 3. *p < 0.05, **p < 0.01 *vs.* Con group. ^#^*p* < 0.05, ^##^*p* < 0.01 *vs.* BHD-M group.

The structure and function of mitochondria were evaluated during CIH exposure. TEM images revealed significant damage to the mitochondrial spines of the myocardial tissue in CIH mice, showing incomplete structure (Figure 3(A)). Mice treated with BHD-M had a relatively complete structure in the mitochondria of the heart than CIH mice. The western blot results revealed that the Opa1 protein level showed a non-significant decrease, and the Drp1 protein level upregulated in the hearts of CIH mice (Figure 3(B,C)), which indicated dysfunctional mitochondrial division. After BHD-M treatment, the elevated Drp1 protein level decreased in CIH mice (Figure 3(B,C)). Subsequently, we examined the mitochondrial pathway-dependent apoptosis of cardiomyocytes exposed to CIH. Similar to previous results, the TUNEL staining images showed that CIH exposure increased the number of apoptotic bodies in cardiomyocytes (Figure 3(D,E)). Concurrently, the ratio of Bcl-2/Bax decreased in the CIH group (Figure 3(F,G)). In addition, induced by CIH, the caspase 3 precursor was cleaved in cardiac tissue (Figure 3(F,G)). However, BHD-M downregulated the number of apoptotic bodies, upregulated the ratio of Bcl-2/Bax and inhibited caspase 3 cleavage in the hearts of mice subjected to CIH (Figure 3(D–G)). Collectively, the aforementioned evidence illustrated that intervention with BHD-M remarkably attenuated the mitochondrial dysfunction and mitochondrial-dependent apoptosis induced by CIH.

### BHD enhanced cardiac autophagy to resist cardiac injury induced by CIH

**Figure 4.**
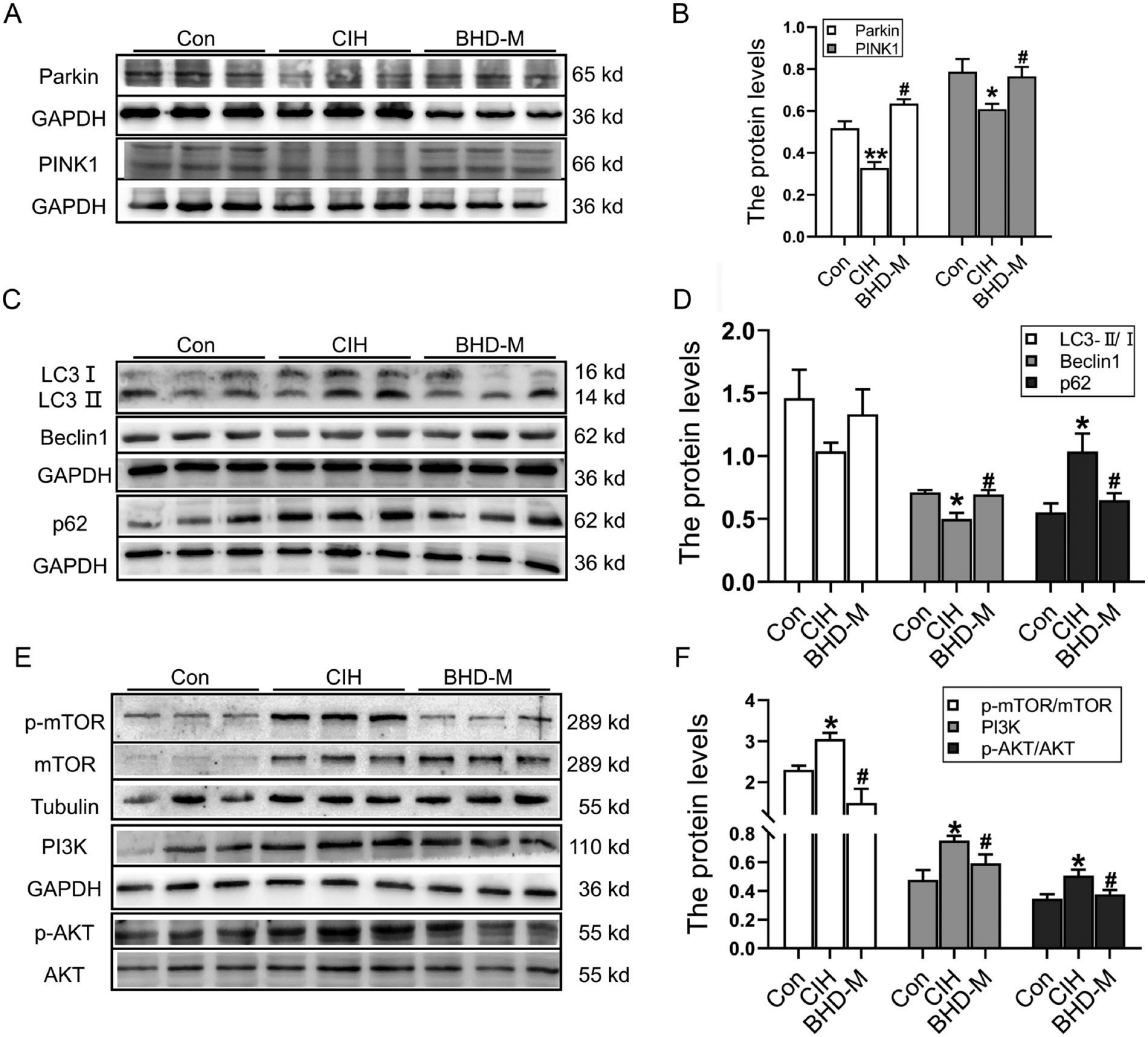
Cardiac autophagy and the PI3K/AKT/mTOR signalling pathway after exposure to CIH. (A,B) The expression of Parkin and PINK1 proteins in heart tissue by western blot. (C,D) The expression of LC3B, Beclin-1, p62 proteins in heart tissue by western blot. (E,F) The expression of PI3K/AKT/mTOR signalling pathway in heart tissue by western blot. The data are presented as the mean ± SEM, *n* = 3. *p < 0.05, **p < 0.01 *vs.* Con group. ^#^*p* < 0.05 *vs*. BHD-M group.

To confirm whether autophagy was related to mitochondrial apoptosis and damage, we detected autophagy-related proteins *via* western blot. The results demonstrated that the Parkin and PINK1 proteins, which are markers of mitochondrial autophagy, were decreased in the CIH group (Figure 4(A,B)). The LC3 II/I ratio showed a downwards trend, but there was no difference between the Con and CIH groups (Figure 4(C,D)). The Beclin-1 level decreased, and the p62 level increased in the CIH group, suggesting that CIH exposure inhibited autophagy in the heart (Figure 4(C,D)). After BHD-M treatment, Beclin-1 levels were elevated, and p62 levels were reduced (Figure 4(C,D)). The PI3K/AKT/mTOR pathway was reported to participate in myocardial hypertrophy and a positive autophagy regulator (Song et al. 2015). In addition, we elevated the PI3K/AKT/mTOR signalling pathway. The results revealed that the PI3K protein level and the ratios of p-AKT/AKT and p-mTOR/mTOR significantly increased after CIH exposure, and BHD-M treatment decreased the expression of the above-mentioned proteins (Figure 4(E,F)). Overall, BHD treatment enhanced autophagy to resist heart injuries induced by CIH.

### BHD efficiently inhibited oxidative stress in cardiac tissue induced by CIH

**Figure 5.**
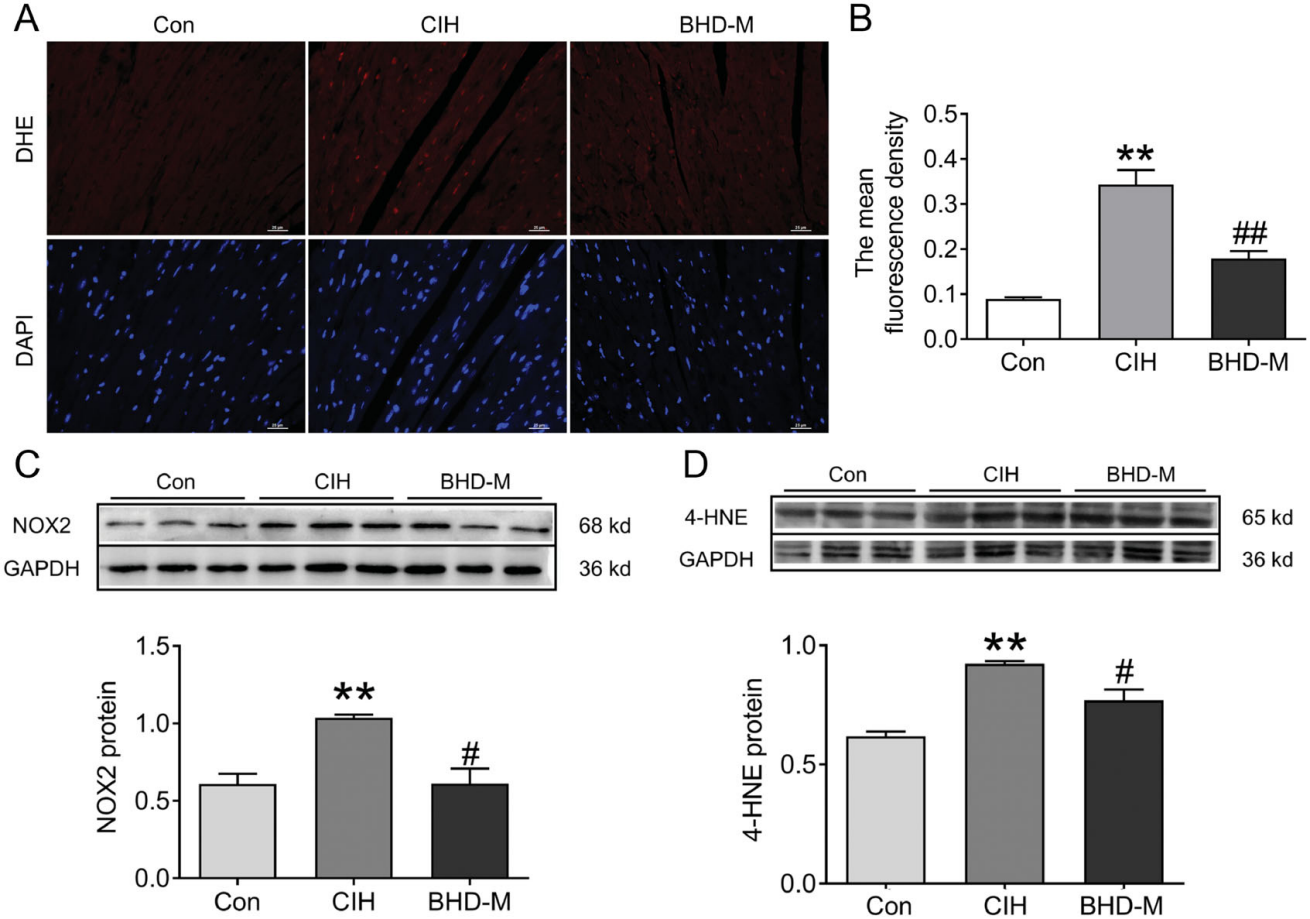
Oxidative stress levels in cardiac tissue subjected to CIH. (A) DHE staining in heart tissue (scale bar = 25 μm, *n* = 3). (B) The mean fluorescence intensity of DHE as shown in panel A. (C,D) The expression and statistics of NOX2 and 4-HNE protein levels. The results are presented as the mean ± SEM, *n* = 3. **p < 0.01 *vs.* Con group. ^#^*p* < 0.05, ^##^*p* < 0.01 *vs.* BHD-M group.

To further clarify whether the cardioprotective effect of BHD was related to the level of oxidative stress during CIH, we sought to determine the ROS content in heart tissue by DHE probe. Compared with the Con group, the mean fluorescence intensity (MFI) significantly increased in the CIH group, and the MFI dropped after BHD-M intervention (Figure 5(A,B)). NADPH oxidase specifically expressed in the mitochondria of cardiomyocytes is a crucial enzyme for catalysing the reduction of oxygen molecules to form superoxide or peroxide (Zhao et al. 2019). Therefore, we measured the expression of NADPH enzymes in the heart and found that CIH exposure elevated the expression of NOX2 (Figure 5(C)). The 4-HNE proteins, a lipid peroxidation product, were elevated in the CIH group (Figure 5(D)). However, NOX2 and 4-HNE could be adjusted reversely by treating with BHD-M administration (Figure 5(C,D)), which suggested that BHD played a vital role in cardiac protection by inhibiting the increase in ROS induced by CIH exposure.

### BHD weakened iron deposits in mitochondria during CIH exposure

**Figure 6.**
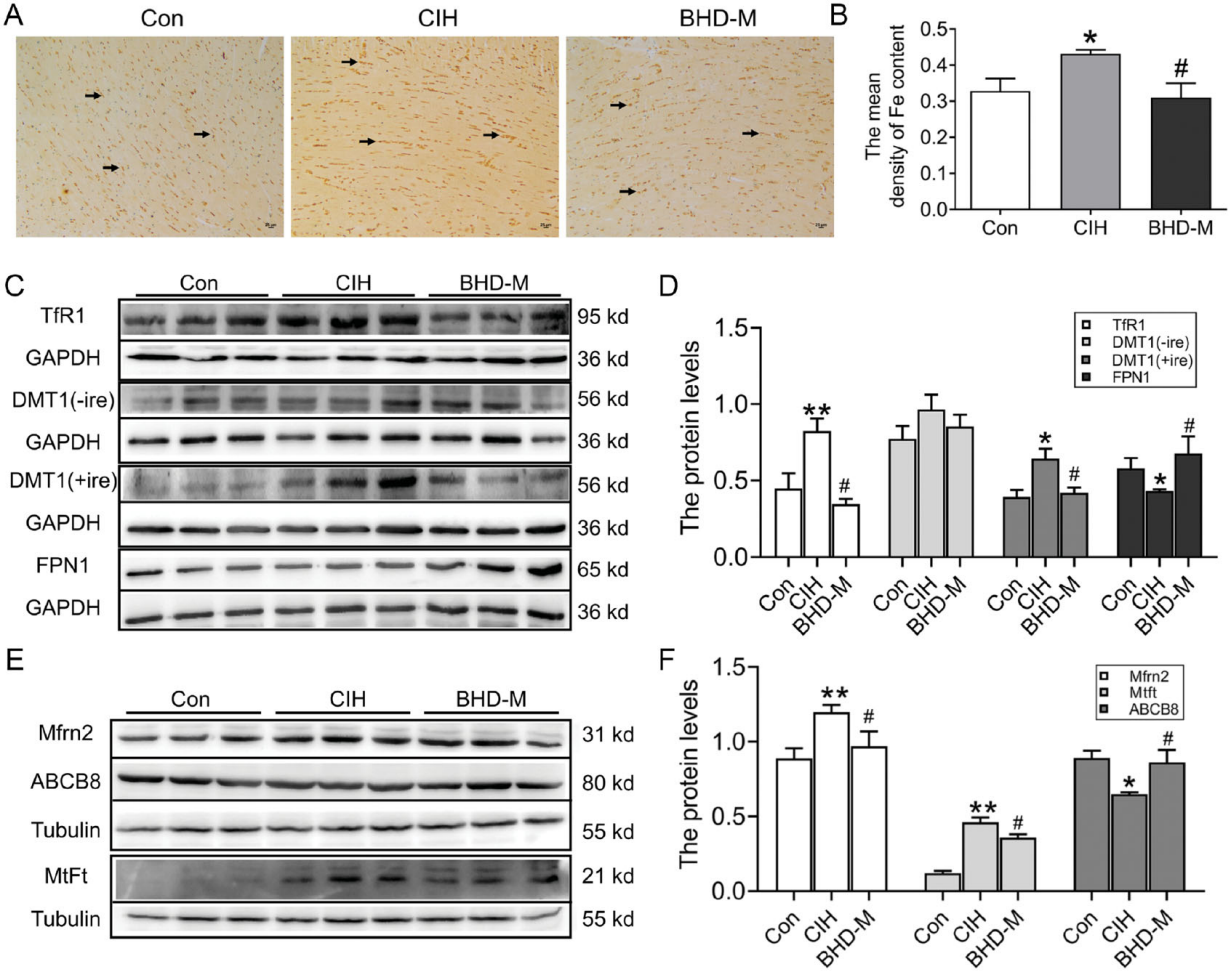
Iron content and iron-related transport proteins in cardiac tissue and mitochondria during CIH. (A) Perls’ staining of heart tissue (scale bar = 25 μm). (B) The mean density of Fe content as shown in panel A. (C,D) The expression and statistics of the iron-related transport proteins, TfR1, DMT1(−ire), DMT1(+ire) and FPN1. (E,F) The expression and statistics of iron transporter-associated proteins in mitochondria, Mfrn2, MtFt and ABCB8. The results are presented as the mean ± SEM, *n* = 3. ∗*p* < 0.05, **p < 0.01 *vs.* Con group. ^#^*p* < 0.05 *vs.* BHD-M group.

Given that excessive iron could accelerate ROS generation, we measured the iron level in heart tissue. Perls’ staining was used to detect the content and distribution of iron. As shown in Figure 6(A,B), the total iron content in the cardiomyocytes of CIH mice increased. The western blot results confirmed that CIH induced the expression of iron uptake proteins, such as TfR1 and DMT1(+ire) in cardiac tissue (Figure 6(C,D)). However, DMT1(−ire) showed no significant difference between the Con and CIH groups (Figure 6(C,D)). Moreover, we found that FPN1, the sole iron release protein, showed a decreasing trend when induced by CIH (Figure 6(C,D)). BHD-M treatment significantly reduced iron deposition by downregulating TfR1 and DMT1(+ire) and upregulating FPN1 expression (Figure 6(C,D)).

After that, we attempted to determine the iron levels in mitochondria. MtFt is ferritin specifically expressed in mitochondria to bind iron (Wu et al. 2016). ABCB8 is implicated in mitochondrial export functions for iron and has roles in the cellular homeostasis of iron and the transport of Fe/S protein precursors (Chang et al. 2016). The western blot results showed that CIH exposure increased the levels of Mfrn2 and MtFt proteins, and reduced the level of the ABCB8 protein (Figure 6(E,F)), indicating iron overload in the mitochondria of cardiomyocytes. After administration of BHD-M, the Mfrn2 and MtFt levels were all declined, but the ABCB8 level was elevated (Figure 6(E,F)). These data indicated that BHD reduced excess iron in mitochondria, which might be the main reason for ROS generation during CIH exposure.

### The main component of BHD could specifically chelate iron in the cardiac tissue of CIH mice

**Figure 7.**
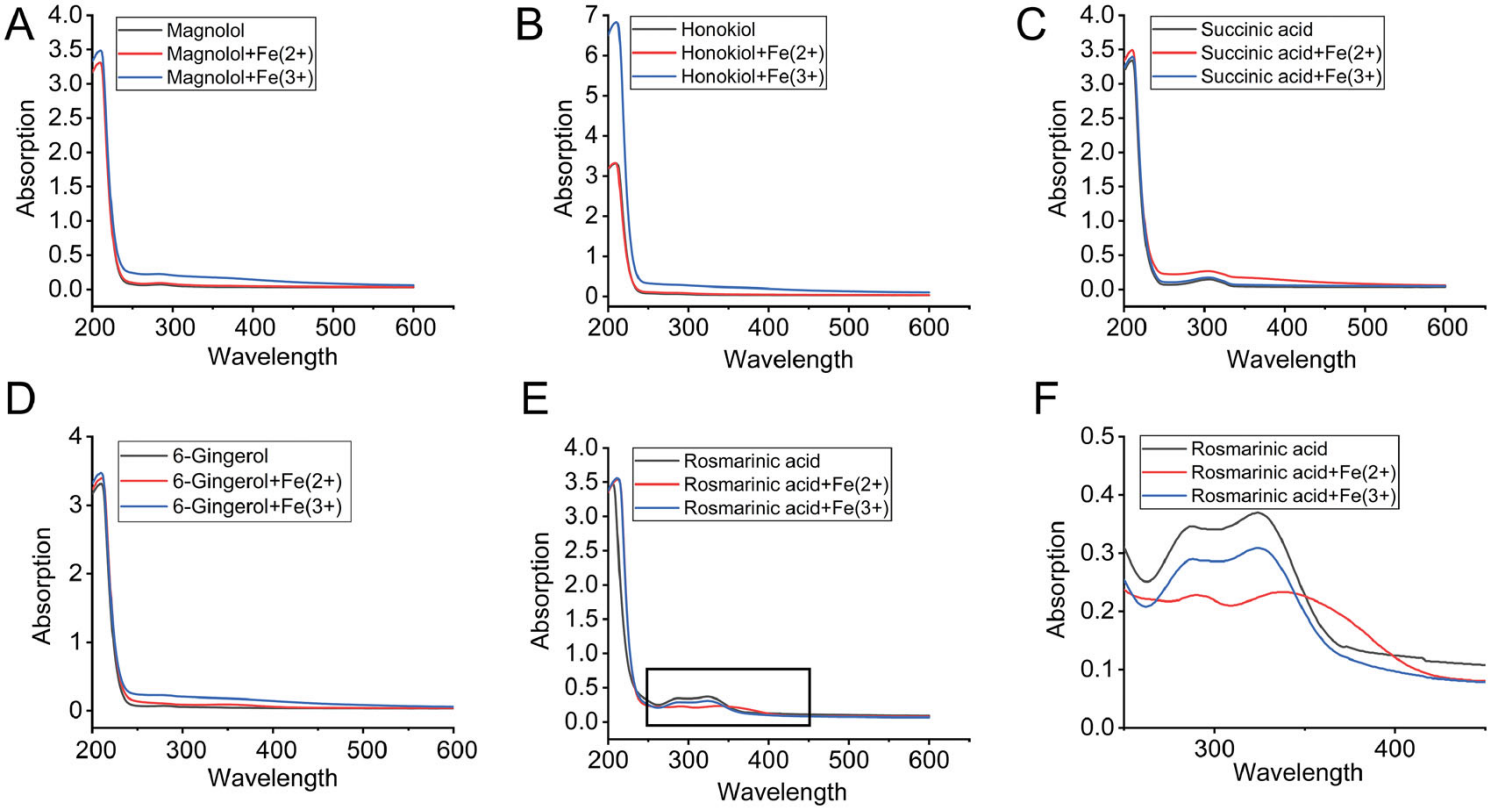
The main component of BHD could specifically chelate iron. The *λ* absorption of (A) Magnolol, (B) Honokiol, (C) Succinic acid, (D) 6-Gingerol, (E) Rosmarinic acid with Fe^2+^ and Fe^3+^, respectively. (F) The enlarged images as shown in panel E.

To further investigate how excess iron was changed in heart tissue of the CIH + BHD group, we performed a chelating experiment *in vitro*. As shown in Figure 7(A–D), the *λ* absorption spectra before and after reaction with Fe^2+^ and Fe^3+^ were not changed in the presence of magnolol, honokiol, succinic acid or 6-gingerol. The *λ* absorption spectra merely changed in the presence of rosmarinic acid-Fe^2+^ and the rosmarinic acid-Fe^3+^ mixture (Figure 7(E,F)). Therefore, we speculated that rosmarinic acid might be the major contributor to removing free iron and the resistance to toxic damage from iron in the heart when subjected to CIH. However, these results did not rule out the possibility that other substances regulate the redistribution of iron during CIH exposure.

## Discussion

CIH is the major pathological feature of OSA, and it is believed to be an independent risk factor for cardiovascular diseases (Roche et al. 2021). During CIH exposure, decreased FS, increased LVESV and decreased LVEDV could lead to a decline in cardiac output, resulting in premature heart failure (Sun et al. 2014; Zhao et al. 2019). Consistent with other findings (Zhao et al. 2019; Jiang et al. 2020), this study indicated that CIH-exposed mice showed pathological changes and fibrosis, which were also risk factors for cardiac dysfunction. Hence, it is necessary to undertake active prevention and treatment in the early stage of CIH. Our research found that BHD could improve the functional and pathological abnormalities of the heart induced by CIH.

Iron is a critically important transition metal and plays a particular role in maintaining normal mitochondrial function *via* involvement in energy metabolism (Lakhal-Littleton 2019). However, the accumulation of iron might eventually exceed the storage capacity of iron, resulting in the generation of a large labile iron pool (LIP, particularly free Fe^2+^) that aggravates ROS generation and oxidative damage (Kell 2009). The increased LIP has been demonstrated to directly affect excitation-contraction coupling, which is the main reason for diastolic dysfunction in hemochromatosis with systemic iron overload (Oudit et al. 2006). Iron-catalysed ROS are also able to increase the LIP level through the reaction of loosely bound iron with Fe-S clusters or other forms (Paul et al. 2017). Therefore, some iron-chelating agents, such as 2,20-bipyridyl (BPD) may chelate mitochondrial iron to alleviate cardiac I/R damage and ROS level (Nicholson et al. 1997; Chang et al. 2016; Vela 2020). This study indicated that BHD could regulate the expression of mitochondrial iron-related proteins in the heart and chelate the Fe^2+^ and Fe^3+^ levels *in vitro*, particularly the main component of the *Perilla* leaf-rosmarinic acid (Figure 7). The discovery mentioned above made BHD more demonstrative for the regulation of iron and ROS, which represents a promising avenue for exploration.

Mitochondria are the primary source of ROS; simultaneously, ROS also induce mitochondrial dysfunction (Chen and Zweier 2014). Drp1 is the primary executor of mitochondrial division, which maintains its dynamic balance (Dorn and Kitsis 2015; Ban et al. 2017; Lee et al. 2020). Deletion of the *Drp1* gene could lead to mitochondrial swelling (Kamerkar et al. 2018), and overexpression of Drp1 could increase Dox-induced injury in cardiomyocytes by accelerating ROS generation (Zhuang et al. 2021). In our study, enhanced expression of Drp1 was shown in the myocardium of mice exposed to CIH, which might contribute to cardiac apoptosis and dysfunction (Dorn and Kitsis 2015).

**Figure 8.**
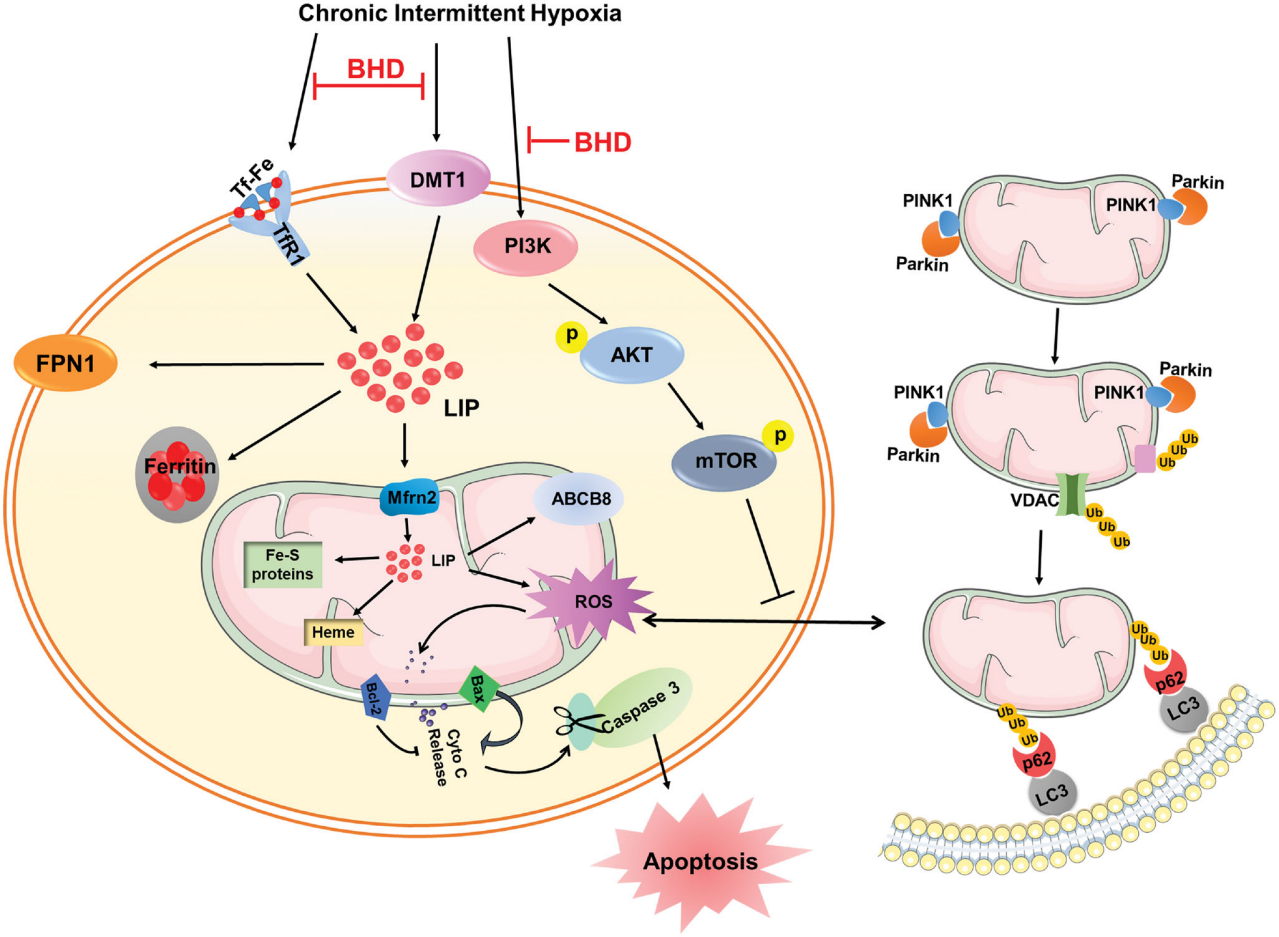
A schematic graph of the proposed cardioprotective mechanism of BHD when exposed to CIH. BHD reduced iron deposition, particularly in mitochondria, by downregulating TfR1, DMT1, Mfrn2 and MtFt, and upregulating FPN1 and ABCB8 expression, and then inhibiting the high level of ROS. BHD enhanced mitochondrial autophagy *via* the PI3K/AKT/mTOR signalling pathway to relieve mitochondrial dysfunction and mitochondrial-dependent apoptosis to exert cardioprotective effects.

Mitophagy is defined as a critical endogenous cellular degradative process responsible for removing damaged mitochondria or mitochondrial proteins (Jahng et al. 2019). Mitochondrial damage, increased ROS or mitochondrial depolarization may lead to the stabilization and accumulation of PINK1 on the outer mitochondrial membrane (OMM), which activates the recruitment of Parkin from the cytosol to the mitochondrial membrane (Sciarretta et al. 2018; Doblado et al. 2021). Once Parkin is recruited or activated, some mitochondrial proteins will be ubiquitinated, conjugate p62, and then recruit the autophagosome membrane *via* LC3, eventually resulting in autophagy (Gottlieb and Thomas 2017; Tang et al. 2019) (Figure 8). Excessive or dysfunctional autophagy leads to an increase in free iron and even regulates cell death, such as ferroptosis (Liu et al. 2020). Iron treatment has been demonstrated to induce autophagy *in vitro* transiently; however, prolonged iron overload would lead to autophagy defects (Jahng et al. 2019). Therefore, it is vital to maintain the dynamic balance of iron in response to stress damage.

Inhibiting mitochondrial autophagy could lead to mitochondrial pathway-dependent apoptosis (Dorn and Kitsis 2015; Gao et al. 2021). Studies have confirmed that the earliest genes involved in apoptosis are the Bcl-2 family, which contains Bcl-2 and Bax (Kalkavan and Green 2018). Bax forms a heterodimer with Bcl-2 *in vivo*, thereby offsetting or reducing the apoptosis-inhibiting effect of Bcl-2, promoting mitochondrial damage and inducing cell apoptosis (Kvansakul and Hinds 2015). During stimulation from I or hypoxia, the outer membrane of the mitochondria is destroyed, which leads to the release of cytochrome C (Cyt-C) originally presented in mitochondria into the cytoplasm (Figure 8) (Brentnall et al. 2013). Cyt-C then combines with apoptosis protease activator-1 (Apaf-1), a caspase-9 precursor, to form an apoptotic complex that activates caspase-9, subsequently activating caspase-3 protein and eventually resulting in the activation of endonucleases in the nucleus for DNA cleavage and apoptosis (Cao et al. 2011; Lang and Hoffmann 2012). Bcl-2-interacting protein 3 (BNIP3) could interact with PINK1 directly and then recruit Parkin, the BNIP3-PINK1 complex involved in Drp1-mediated mitochondrial fission (Doblado et al. 2021). Simultaneously, it has been reported that phosphorylation of Akt participates in Bax-mediated apoptosis under H_2_O_2_ exposure (Sadidi et al. 2009). Our results revealed a lower ratio of Bcl-2/Bax and a higher level of cleaved-caspase 3, which indicated CIH exposure-induced mitochondrial pathway-dependent apoptosis (Figure 3). All in all, BHD exerted cardiac protection by inhibiting apoptosis.

The myocardial protective effect of BHD is closely related to its antioxidant activity, including that of the decoction mixture or its main components. Magnolol and honokiol are reported to eliminate oxygen consumption and MDA production in isolated rat heart mitochondria induced by FeSO_4_ (Lo et al. 1994). Magnolol could treat melanoma by the PI3K/Akt pathway (Emran et al. 2019) and attenuates prefrontal cortex oxidative stress injury in CMS mice (Cheng et al. 2018). Rosmarinic acid has been proven not only to improve doxorubicin-induced cardiotoxicity (Rahbardar et al. 2021) but also to chelate labile iron and improve DNA damage induced by H_2_O_2_ (Gerogianni et al. 2018). Therefore, combined with our chelating experiments, we speculated that rosmarinic acid might be used as a chelator to reduce iron toxicity damage.

## Conclusions

Our experimental results revealed that an excess of iron in myocardial cells might be involved in oxidative stress and mitochondrial dysfunction when exposed to CIH. BHD could specifically decline mitochondrial iron to improve heart dysfunction and structural damage caused by CIH. These findings provide the further theoretical basis for the use of TCM for cardiovascular injury in OSA patients.

## Data Availability

The data used to support the findings of this study are included within the article.
